# Drought disrupts quinoa seed germination and seedling establishment via sugar metabolism and hormonal perturbations

**DOI:** 10.1186/s12870-026-09410-z

**Published:** 2026-06-30

**Authors:** Yanmei Gao, Weiwei Chen, Jie He, Pengrui Feng, Yating Wang, Min Sun, Zhiqiang Gao, Zhimin Wang, Yongqing Zhang, Meng Zhang

**Affiliations:** 1 School of Life Science, Shanxi Normal University, Taiyuan, 030031 China; 2 College of Life Sciences, Sichuan University, Chengdu, 610065 China; 3 College of Agronomy and Biotechnology, China Agricultural University, Beijing, 100193 China; 4 College of Agriculture, Shanxi Agricultural University, Taigu, 030801 China

**Keywords:** Quinoa, Drought stress, Phytohormone, Sugar metabolism, Antioxidant system

## Abstract

Drought stress inhibits quinoa (*Chenopodium quinoa* Willd.) seed germination and seedlings growth, significantly affecting yield and quality. Nevertheless, the molecular mechanisms underlying quinoa’s drought stress response are not yet fully elucidated. Therefore, a multi-omics analysis of the response to drought stress was conducted using two drought-resistant quinoa varieties (LL1 and LQ18) subjected to normal (CK) and severe (DS2, 20% PEG-6000) drought conditions. Drought stress exerted no statistically significant impact on germination rate, germinative force, and germination index for LQ18, but DS2 treatment reduced these germination indicators for LL1. Concomitantly, DS2 treatment notably decreased seedlings height, hypocotyl length, radicle length, dry and fresh weight, especially for LL1. Multi-omics analyses revealed that DS2 treatment markedly downregulated genes related with starch and sucrose metabolism and galactose metabolism during the radicle emergence stage, whereas these pathways were subsequently upregulated at the cotyledon expansion stage. Thus, the starch and sucrose content increased at the two stages, while glucose content decreased at radicle emergence stage but increased at cotyledon expansion stage. The low glucose concentration at radicle emergence stage likely suppressed abscisic acid (ABA) biosynthesis, resulting in no significant change in the abundance of the differentially accumulated metabolites (DAM) of ABA. The hormonal balance was significantly altered, with the gibberellins (GAs)/ABA ratio being significantly lower in LL1 under DS2, while it was slightly up-regulated in LQ18. In addition, DS2 treatment induced genes expression of antioxidant systems, in which soluble sugars, proline, and malondialdehyde content accumulated, and raffinose, stachyose, trehalose, glutathione, kaempferol, and quercetin abundance increased, especially for LQ18. Collectively, the findings of this study contribute to a deeper understanding of the drought tolerance mechanisms of quinoa, thereby providing a theoretical foundation for future research on quinoa’s drought resistance.

**Supplementary Information:**

The online version contains supplementary material available at https://doi.org/10.1186/s12870-026-09410-z.

## Introduction

In recent years, quinoa (*Chenopodium quinoa* Willd.) has garnered significant global attention in agriculture and food research due to its high nutritional value and numerous potential health benefits [1, 2]. Therefore, quinoa has also been occasionally considered a “*superfood*”, “*golden grain*”, and “*perfect strategic food*” [1]. Moreover, quinoa is extremely adaptable and resilient, and can survive in harsh environments such as cold, drought, low temperatures and salinity [3, 4]. In China, quinoa is predominantly cultivated in water scarcity regions–including Shanxi, Inner Mongolia, Gansu, Qinghai–where natural precipitation serves as the primary water source. The average annual rainfall in these areas ranges is about 50ཞ500 mm, with 60ཞ80% concentrated from June to September [5]. This seasonal precipitation pattern does not align with the typical quinoa sowing period (April–May), severely affecting seed germination and seedlings growth of quinoa. Therefore, further research into quinoa’s responses to drought stress during these early developmental stages is of considerable practical significance for enhancing its drought tolerance.

Seed germination is a critical and complex regulatory phase in plant growth, modulated by diverse physical and chemical environmental cues [6]. The transition from a dry seed to visible radicle protrusion involves water uptake and activates processes such as DNA repair, cell wall metabolism, phytohormone signal transduction, and the synthesis of amino acid, protein, and transport and nucleic acid [6–9]. During post-germination, hypocotyl elongation and cotyledon expansion occur alongside the activation of photosynthesis and energy metabolism, collectively promoting seedling establishment [6, 8]. Drought stress during seed germination and early growth seriously affects early development, growth patterns, and survival rates, ultimately compromising agricultural productivity.

Under adverse conditions such as drought, seed storage materials play a vital protective role. Quinoa seeds are rich in starch and dietary fiber. To sustain germination and early seedling growth under drought conditions, sugars are catabolized to generate ATP, thereby fueling these critical energy-demanding processes [10]. Seeds development in quinoa predominantly enriches metabolic pathways such as galactose metabolism, pentose and glucuronate interconversions, amino acid biosynthesis, and carbon metabolism, with significant accumulation of metabolites like raffinose and phosphoenolpyruvate [8, 11]. Consistently, under drought conditions, the contents of raffinose family oligosaccharides (RFOs), mannose, glucose, and fructose increase significantly [12, 13]. Starch and sucrose metabolism provides energy during the transition from germination to radicle elongation. As cotyledons expand, photosynthesis is fully activated, further supporting seedling growth [8]. Phytohormones, key signaling molecules mediating environmental responses, are associated with the regulation of seed germination. Among them, ABA and GAs are two of the most significant hormones in this process. ABA promotes and sustains seed dormancy while suppressing germination, while GAs act antagonistically to promote it [9, 14]. The equilibrium between GAs and ABA acts as a central regulatory node, controlling not only germination potential but also extending its influence to plant development and stress adaptation [15, 16].

A primary consequence of drought stress in seeds is the excessive buildup of reactive oxygen species (ROS). The excessive accumulation of ROS perturbs cellular redox balance, leading to persistent oxidative damage in essential biomacromolecules including nucleic acids, lipids, and proteins. This, in turn, impairs normal seed germination and subsequent seedling establishment [12, 17]. To mitigate oxidative damage and sustain metabolism under ROS stress, plants employ a sophisticated antioxidant system; this defense includes upregulating enzymes like SOD and POD while increasing non-enzymatic antioxidants such as glutathione and betalain. These coordinated actions collectively scavenge excess ROS and alleviate oxidative stress [18–22]. Beyond enhancing antioxidant capacity, seeds adapt to drought by regulating cellular osmotic potential by synthesizing and accumulating compatible solutes including proline, glycine betaine, and soluble sugars. These substances effectively reduce cellular osmotic pressure, improve water retention and uptake, and help sustain cellular activity under water deficit [12, 23]. Furthermore, secondary metabolites produced via the phenylpropanoid metabolic pathway, including flavonoids and lignin derivatives, also contribute to drought adaptation, further enriching the plant’s drought adaptation strategies [20, 24, 25]. Therefore, research into quinoa’s drought response mechanisms in early growth stages is of direct relevance, as it informs strategies to improve crop resilience and fosters sustainable industry expansion.

Recent multi-omics studies have substantially advanced our understanding of drought tolerance mechanisms in quinoa. Transcriptomic analyses indicate that drought stress triggers the upregulation of key genes, including those in ABA signaling, osmolyte biosynthesis (e.g., proline and betaine), and stress-related transcription factors like AP2/ERF, NAC, MYB, and WRKY, that collectively regulate the plant’s adaptive response [22, 26]. Untargeted metabolomic studies further illuminated concomitant metabolic adjustments, highlighting the accumulation of compatible solutes for osmotic adjustment, and flavonoids and other phenolic compounds crucial for antioxidant defense [27–29]. The combined application of transcriptomics and metabolomics has underscored the relevance of α-linolenic acid metabolism, starch and sucrose metabolism, and flavonoid biosynthesis pathways in improving quinoa’s resilience under drought conditions [26, 27]. However, most current researches have focused on the seedling and flowering stages, leaving a critical knowledge gap regarding integrated physiological, transcriptomic, and metabolomic responses during seed germination.

Polyethylene glycol (PEG-6000), a high-molecular-weight polymer, is widely used to simulate drought stress by lowering the water potential of growth media, enabling precise quantification and replication under controlled growth conditions [12]. To elucidate the regulatory networks governing quinoa’s drought responses during seed germination and seedling establishment, we conducted an integrated phenotypic, physiological, transcriptomic and metabolomic analyses. These focused on key pathways including starch/sucrose metabolism, galactose metabolism, plant hormone signaling, betalain biosynthesis, and phenylpropanoid/flavonoid biosynthesis. Our findings elucidate novel aspects of drought tolerance mechanisms in early quinoa development, thereby laying a foundation for designing strategies to enhance crop resilience under water-limited conditions.

## Materials and methods

### Experimental design

Two drought-tolerant quinoa varieties, LL1 and the novel breeding line LQ18, were obtained from the Gansu Academy of Agricultural Sciences (GAAS) (Lanzhou, China). Uniform, plump seeds free of pests and diseases were surface-sterilized in 10% NaClO (Tianjin Kemiou Chemical Reagent Co., Ltd., Tianjin, China) for 15 min, followed by 3–5 rinses with sterile water. For germination assays, five replicates were established per variety and treatment. Each replicate was prepared by arranging 100 seeds in an 11 cm disposable Petri dish containing two layers of filter paper. Drought stress was simulated using PEG-6000 (Tianjin Kemiou Chemical Reagent Co., Ltd., Tianjin, China). Preliminary experiments were conducted with LL1 under varying levels of drought stress (Supplementary Table 1). To simulate drought stress, Petri dishes were treated with 10 mL of aqueous PEG-6000 solutions at concentrations of 2.5%, 5%, 10%, 15%, and 20%, while the control (CK) group received an equal volume of sterile water. All dishes were initially kept in complete darkness at 25 °C for 3 days to simulate the subterranean environment during early imbibition and radicle protrusion. Thereafter, dishes were transferred to a 16 h light/8 h dark photoperiod to mimic the transition to post-germination seedling establishment, promote cotyledon greening, and avoid etiolation. The PEG-6000 solution was changed daily to maintain a stable drought environment. Germinated seeds were recorded at 4 h intervals until no longer germination occurred. Germination was defined as radicle length greater than or equal to 2 mm. Based on the germination rates (Supplementary Table 1), 5% and 20% PEG-6000 were selected as mild (DS1) and severe (DS2) drought stress treatments, respectively. To further evaluate germination characteristics, seed germination and seedling growth experiments were conducted using the LL1 and LQ18 varieties.

### Sampling and measurements

The germination rate was determined on day 7 as the proportion of germinated seeds relative to the total seeds tested. Germinative force, measured on day 3, was expressed as the percentage of seeds that had germinated by that time relative to the total. Additionally, the germination index was evaluated based on the cumulative percentage of seeds germinated on each respective day (*i*) [30].

**Fig. 1:**
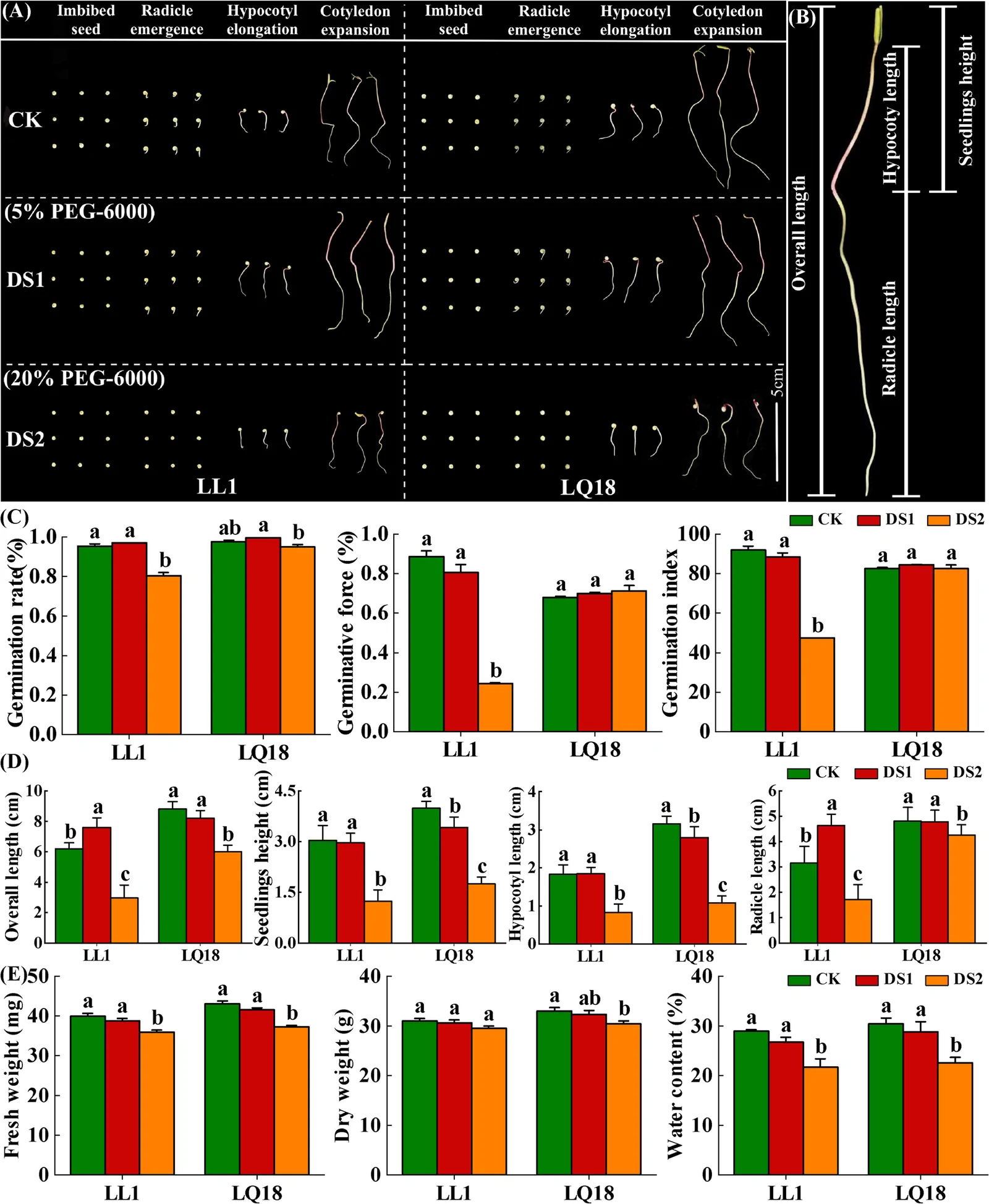
Seed germination characteristics and seedling phenotype of two quinoa varieties (LL1 and LQ18) in the CK (control), DS1 (mild drought stress, 5% PEG-6000) and DS2 (severe drought stress, 20% PEG-6000) treatments. **(A)** Four stages of quinoa seeds from water imbibition to seedling emergence. It shows the germination and growth status of quinoa under 5% and 20% PEG-6000 treatments at the point when the CK had reached the corresponding stage. **(B)** Schematic diagram of the length of each part of the quinoa seedlings. **(C)** Seed germination characteristics including germination rate, germinative force, and germination index. **(D)** The length of each part of the quinoa seedlings. **(E)** Fresh and dry weight of quinoa. Different lowercase letters indicate significant differences between drought stress treatments in the same variety. Bars mean SE (*n* = 3)

At the radicle emergence and cotyledon expansion stages, samples were collected based on these specific morphological landmarks rather than at fixed time points (as depicted in Fig. 1A), to ensure that the same developmental phase was compared. The radicle emergence stage was defined as visible radicle protrusion from the seed coat (germination criterion: radicle length ≥ 2 mm), whereas the cotyledon expansion stage was defined as the stage at which cotyledons were visibly expanded following hypocotyl elongation. At cotyledon expansion stages, ten seedlings per replicate were sampled to measure seedlings height, radicle length, and hypocotyl length (Fig. 1B). Each treatment was repeated three times. Subsequently, seedlings were rinsed five times with deionized water, then surface water was blotted out with absorbent paper. Fresh weight was recorded immediately after harvest. Dry weight was measured by subjecting the samples to oven drying at 60 °C for 48 h. Water content was derived by applying the following formula:

1 $$Water\;content\:\left(\%\right)=(Fresh\;weight-Dry\;weight)/Dry\;weight\times100$$

At the radicle emergence and cotyledon expansion stages, more than 200 seeds or seedlings were sampled per replicate. Samples were rinsed five times in deionized water, enclosed in aluminum foil, snap-frozen in liquid nitrogen, and then transferred to -80 °C storage for subsequent RNA isolation, untargeted metabolomics, as well as assays of sugar content, osmotic substances, and antioxidant activity.

### RNA-Seq analysis

Total RNA was isolated from germinating seeds and seedlings collected during the radicle emergence and cotyledon expansion stages under both CK and DS2 treatments, utilizing TRIzol™ Reagent (Invitrogen, CA, USA) by Novogene Co., Ltd. (Beijing, China). Following extraction, RNA integrity was confirmed by employing an RNA Nano 6000 Assay Kit (Agilent Technologies, CA, USA) on a Bioanalyzer 2100 system (Agilent Technologies, CA, USA). The RIN values of all RNA-seq samples ranged from 8.1 to 9.8, indicating that all samples met the quality requirements for library construction and sequencing. Detailed RIN values are provided in Supplementary Table 2. mRNA was subsequently isolated from total RNA through poly-A Oligo (dT) magnetic bead-based enrichment. Following cDNA library construction, PCR products underwent purification using an AMPure XP system (Beckman Coulter, Brea, CA, USA). Prior to 150-bp paired-end sequencing on an Illumina Novaseq platform, final library quality was verified with an Agilent Bioanalyzer 2100 (Agilent Technologies, CA, USA). Raw sequencing reads were then processed to eliminate adapter sequences, poly-N stretches, and low-quality bases, resulting in clean data. Using the HISAT2 aligner, the clean reads were aligned to the quinoa reference genome (assembly: ncbi_chenopodium_quinoa_gcf_001683475_1_asm168347v1). Transcript assembly was performed using StringTie (v1.3.3b) with reference-based mode. Transcript abundance was assessed in FPKM (fragments per kilobase million). Differential expression analysis, conducted using the DESeq2 package (v1.20.0) in R (v4.4.2) with thresholds of |log_2_(fold change)| ≥1 and adjusted p-value < 0.05, identified DEGs. Their functional profiling was performed using the Gene Ontology (GO) and Kyoto Encyclopedia of Genes and Genomes (KEGG) databases. Inter-sample correlations were computed via the cor() function in R (v4.4.2) and correspondingly visualized. GO term enrichment analysis for DEGs was executed on the agriGO v2.0 platform using the Singular Enrichment Analysis tool, with terms exhibiting an FDR < 0.05 (hypergeometric test) deemed significantly enriched. Finally, K-means clustering (k = 6, determined by the elbow method) was applied to Z-score normalized FPKM values of DEGs using the kmeans() function in R.

### Metabolome measurement

Metabolomic profiling was conducted using an untargeted approach. Samples were analyzed via ultra-high performance liquid chromatography coupled with high-resolution tandem mass spectrometry (UHPLC-LC-MS/MS) by Novogene Co., Ltd. (Beijing, China). Raw mass spectrometry files (.raw) were processed in Compound Discoverer 3.1 software (Thermo Fisher Scientific, Waltham, MA, USA) for spectral analysis, database searching, and metabolite qualification/quantification, followed by rigorous quality control. Multivariate statistical analyses, principal component analysis (PCA) and orthogonal partial least squares-discriminant analysis (OPLS-DA), were applied to distinguish metabolic patterns between groups. Differentially accumulated metabolites (DAMs) were screened using thresholds of VIP > 1.0, |log_2_(fold change) | > 0.26 (equivalent to FC > 1.2 or < 0.83), and adjusted p-value < 0.05. Identified DAMs were functionally annotated and subjected to pathway enrichment analysis via KEGG mapping.

### Integrated transcriptomic and metabolomic analysis

The nine-quadrant association analysis was performed by calculating Pearson correlation coefficients between the normalized expression levels of all DEGs and the relative abundances of all DAMs. Associations with an absolute correlation coefficient (|r|) greater than 0.8 and a P-value below 0.05 were deemed statistically significant. All analyses and corresponding visualizations were conducted using in-house R scripts (v 4.4.2). Subsequently, mapping of all identified DEGs and DAMs to the KEGG pathway database was carried out, aimed at elucidating the key biological pathways jointly influenced by both transcriptional and metabolic changes.

### Quantitative real-time PCR (qRT-PCR) analysis

The genes selected for RT-qPCR validation were representative DEGs from major drought-responsive pathways identified by the integrated transcriptomic and metabolomic analyses, and each pathway included genes showing significant up- or down-regulation under drought stress. RT-qPCR validation was conducted independently in our laboratory using newly extracted RNA. Total RNA extraction was performed using the TransZol Up Plus RNA Kit (TransGen Biotech, Beijing, China) according to the supplier’s instructions. Subsequently, first-strand cDNA was synthesized from the purified RNA with the One-Step gDNA Removal and cDNA Synthesis Supermix (TransGen Biotech, Beijing, China). Gene-specific primers were designed employing the NCBI Primer-BLAST online tool. qRT-PCR was carried out on a Bio-Rad CFX96 Real-Time PCR Detection System (Bio-Rad, Hercules, CA, USA). Each reaction (20 µL total volume) contained 10 µL of 2× TransStart Top Green qPCR SuperMix (TransGen Biotech, Beijing, China), 0.5 µL each of forward and reverse primers (10 µM), 1 µL of cDNA template, and 8.0 µL of nuclease-free water. The thermal cycling program was as follows: initial denaturation at 95 °C for 3 min, followed by 40 cycles of 95 °C for 15 s and 60 °C for 1 min. A melting curve analysis was performed from 65 °C to 95 °C to verify primer specificity. Amplification data were analyzed using Bio-Rad CFX Maestro software (version 2.2; Bio-Rad, Hercules, CA, USA). The quinoa *Actin-1* gene was used as an internal reference gene for normalization, based on its stable expression under abiotic stress-related conditions in previous published studies [31]. The relative expression of target genes for each treatment was normalized to the corresponding control and determined via the 2^−ΔΔCt^ method. Three independent biological replicates, each run-in technical triplicate, were analyzed. Primer sequences used in qPCR are provided in Supplementary Tables 3 and detailed amplification efficiency data are presented in Supplementary Table 4.

### Measurement of sugar, starch and soluble protein content

To determine soluble sugar, sucrose, and starch contents, approximately 100 mg of oven-dried sample powder was first extracted with 8 mL of deionized water at 100 °C for 20 min. The resulting mixture was cooled to room temperature and centrifuged at 4,500 rpm for 10 min. The supernatant was collected and reacted with anthrone reagent (Tianjin Kemiou Chemical Reagent Co., Ltd., Tianjin, China) for soluble sugar quantification at 620 nm. NaoH (Tianjin Fengchuan Chemical Reagent Technology Co., Ltd., Tianjin, China), HCL (Tianjin Yongda Chemical Reagent Co., Ltd., Tianjin, China) and resorcinol (Aladdin Biochemical Technology Co., Ltd., Shanghai, China) were added to the supernatant to determine sucrose content at 480 nm. The insoluble residue was then hydrolyzed by boiling with 7 mL of 3 mol L^–1^ HCl at 100 °C for 45 min. After cooling and centrifugation at 4,000 rpm for 15 min, the supernatant was neutralized with 7 mL of 3 mol L^–1^ NaOH. Starch content was subsequently determined via enzymatic hydrolysis of the residue to glucose, followed by spectrophotometric measurement at 620 nm. Glucose concentration was determined at 505 nm using commercial assay kits (BC2500, Solarbio, Beijing, China) on a visible-light spectrophotometer (INESA Analytical Instrument Co., Ltd, Shanghai, China).

Soluble protein content was determined by first homogenizing approximately 100 mg of frozen tissue in 1 mL of distilled water, followed by centrifugation at 10,000 rpm for 10 min. Then, 0.1 mL of the supernatant was combined with 5 mL of Coomassie Brilliant Blue G-250 solution (Tianjin Guangfu Fine Chemical Research Institute, Tianjin, China) and mixed thoroughly by vortexing (Jiangsu Jintan Ronghua Instrument Manufacturing Co., Ltd., Jiangsu, China). After a 2-minute incubation period, absorbance was recorded at 595 nm using a spectrophotometer, and protein concentration was calculated based on a standard curve.

### Analysis of antioxidant properties

Malondialdehyde (MDA) content was quantified using the thiobarbituric acid (TBA) method, in which the absorbance of the MDA–TBA adduct was measured spectrophotometrically as previously described [32]. Proline content was analyzed by the ninhydrin reaction under acidic conditions, with the resulting red chromophore detected spectrophotometrically at 520 nm [33]. For enzyme activity assays, frozen tissue was homogenized in an extraction buffer and centrifuged to obtain the crude enzyme extract. Superoxide dismutase (SOD) activity was evaluated by measuring its ability to inhibit the photochemical reduction of nitroblue tetrazolium (NBT) at 560 nm, whereas peroxidase (POD) activity was determined by monitoring guaiacol oxidation at 470 nm [34]. Total flavonoid content was measured using a colorimetric method with aluminum chloride, and the absorbance was read at 510 nm [35].

### Statistical analyses

For data meeting both normality and homogeneity of variance assumptions, a two-way ANOVA was carried out in SPSS 20.0 (IBM Corp., Armonk, NY, USA), followed by post-hoc LSD tests at either the 95% or 99% confidence level. When these assumptions were not satisfied, data were either subjected to transformation (log_10_ or square root) or analyzed with non-parametric tests in R (v4.4.2). For significant Kruskal-Wallis results, multiple comparisons were performed using Dunn’s Test at the specified confidence levels (95% or 99%). All graphical presentations were created using Origin Pro 2019 (OriginLab Corporation, Northampton, MA, USA) and Adobe Illustrator CC 2017 (Adobe Inc., San Jose, CA, USA).

## Results

### Seed germination characteristics and seedling phenotype under drought stress

Seed germination is initiated by rapid water uptake from dry seeds until the protrusion of radical. Subsequently, a continuous increase in fresh weight and seed size prompted the radicle emergence. Ultimately, hypocotyl elongation and cotyledon expansion promote seedling establishment (Fig. 1A). ANOVA shown that drought stress and genotype had significant effects on the germination indicators (germination rate, germinative force, and germination index), seedling phenotype, and biomass. Their interaction also significantly influenced germination indicators and seedling phenotype (Supplementary Table 5). At the radicle emergence stage, drought stress did not affect germination rate, germinative force, and germination index in LQ18, but severe drought stress (DS2, 20% PEG-6000) reduced these indicators in LL1 (Fig. 1C). At the cotyledon expansion stage, we measured the length of each part of the seeding (Fig. 1B). Under control (CK) conditions, LQ18 had significantly higher seedling height, hypocotyl length, and radicle length than LL1, resulting in longer overall length (Fig. 1D). Compared with CK, mild drought stress (DS1, 5% PEG-6000) had no effect on seedling height and hypocotyl length in LL1, but significantly increased radicle length, leading to increased overall length. In contrast, DS1 decreased seedling height and hypocotyl length in LQ18, with no significant effect on radicle length and overall length (Fig. 1D). Under DS2, both varieties showed notable reductions in all seedling dimensions relative to CK. In LL1, seedling height and radicle length decreased by 59.3% and 45.8%, respectively; in LQ18, the corresponding reductions were 56.1% and 11.7% (Fig. 1D). Consequently, DS2 treatment led to marked reductions in fresh weight, dry weight, and water content (Fig. 1E). These results indicate that LQ18 exhibits stronger drought resistance than LL1, and DS2 markedly suppressed seed germination and seedling growth. 

### Transcriptome analysis of differentially expressed genes (DEGs) under DS2 vs. CK

**Fig. 2:**
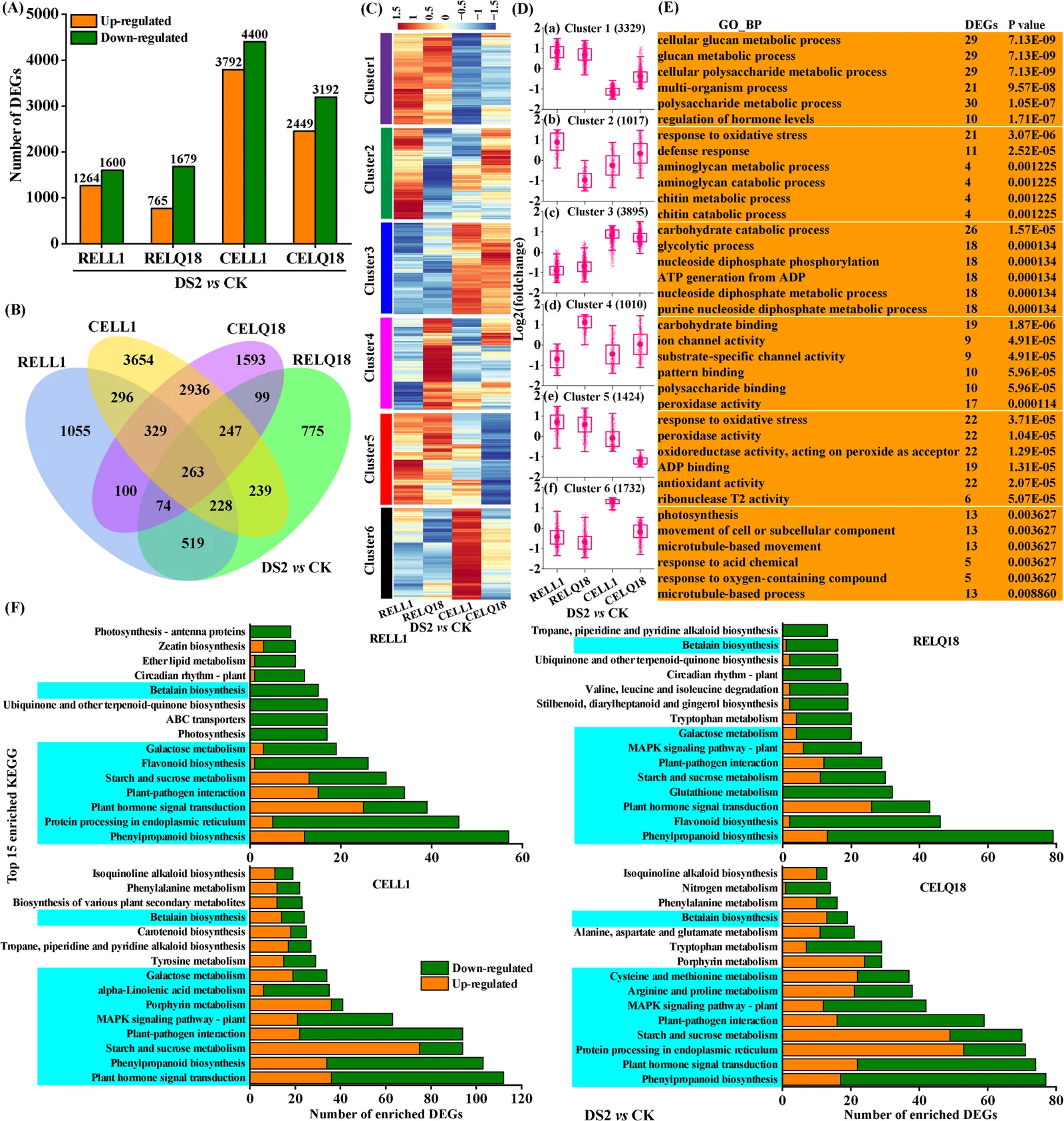
Numbers and categories of differentially expressed genes (DEGs) for quinoa seedlings in DS2 vs. CK at RELL1 (LL1 at radicle emergence), RELQ18 (LQ18 at radicle emergence), CELL1 (LL1 at cotyledon expansion), and CELQ18 (LQ18 at cotyledon expansion). **(A)** The numbers of the up-regulated and down-regulated DEGs for quinoa seedlings. **(B)** Venn diagram showing the DEGs among four comparations in DS2 vs. CK at RELL1, RELQ18, CELL1, and CELQ18. **(C)** *K*-means clustering of DEGs. **(D)** a-f, expression patterns of genes specific to different varieties and periods. **(E)** The GO enrichment analysis of genes in each cluster, with *P* values and GO category. **(F)** The top 15 enriched KEGG pathways of the enriched DEGs in DS2 vs. CK. The cyan-highlighted represents the most enriched KEGG

To elucidate the drought response mechanisms in two quinoa varieties, RNA sequencing was conducted on seedlings at radicle emergence and cotyledon expansion stages. Pearson correlation coefficients between samples exceeded 0.92, indicating high reproducibility (Supplementary Fig. S1A). PCA revealed clear separation between drought-stressed and control groups, and between the two developmental stages (Supplementary Fig. S1B). Four pairwise comparisons were conducted: RELL1_DS2 vs. RELL1_CK, RELQ18_DS2 vs. RELQ18_CK, CELL1_DS2 vs. CELL1_CK, CELQ18_DS2 vs. CELQ18_CK, corresponding respectively to the two genotypes (LL1 and LQ18) at the two developmental stages (radicle emergence and cotyledon expansion) under severe drought stress (DS2) vs. control (CK). For each pairwise comparison, we determined the counts of both up- and down-regulated differentially expressed genes (DEGs). In both developmental periods, down-regulated DEGs significantly outnumbered up-regulated genes in the DS2 vs. CK comparison, with more DEGs identified at cotyledon expansion than at radicle emergence (Fig. 2A).

We further characterized DEGs from the DS2 vs. CK comparison across different growth stages and quinoa varieties (Fig. 2B). We found that a total of 12,407 DEGs and 263 co-DEGs (common across all comparisons) were identified. Shared DEGs were predominantly enriched in carbohydrate metabolism-related biological processes (Fig. 2B, Supplementary Fig. S2A). To further identify the changes of gene expression profiles, all these 12,407 DEGs between DS2 and CK treatments were divided into clusters (*k* = 6) by *k*-means analysis (Fig. 2C-D). Cluster 1st and 5th (4753 DEGs), up-regulated at radicle emergence but down-regulated at cotyledon expansion, and were mainly related to carbohydrate metabolism, hormonal response, and oxidative stress response (Fig. 2D-E). In contrast, the clusters 3rd (3895 DEGs), down-regulated at radicle emergence but up-regulated at cotyledon expansion, and was involved in carbohydrate catabolic, glycolytic, nucleoside diphosphate metabolic process (Fig. 2D-E). Then, we analyzed the top 15 enriched KEGG pathways of the DEGs in the pairwise comparations (RELL1_DS2 vs. RELL1_CK, RELQ18_DS2 vs. RELQ18_CK, CELL1_DS2 vs. CELL1_CK, CELQ18_DS2 vs. CELQ18_CK) and the KEGG of the co-DEGs (Fig. 2F, Supplementary Fig. S2B), and found the pathway “phenylpropanoid biosynthesis”, “plant hormone signal transduction”, “starch and sucrose metabolism”, “galactose metabolism”, and “betalain biosynthesis” were highly enriched at both stages. “Flavonoid biosynthesis” was also enriched at radicle emergence. 

### Metabolomic adjustments under DS2 vs. CK

**Fig. 3:**
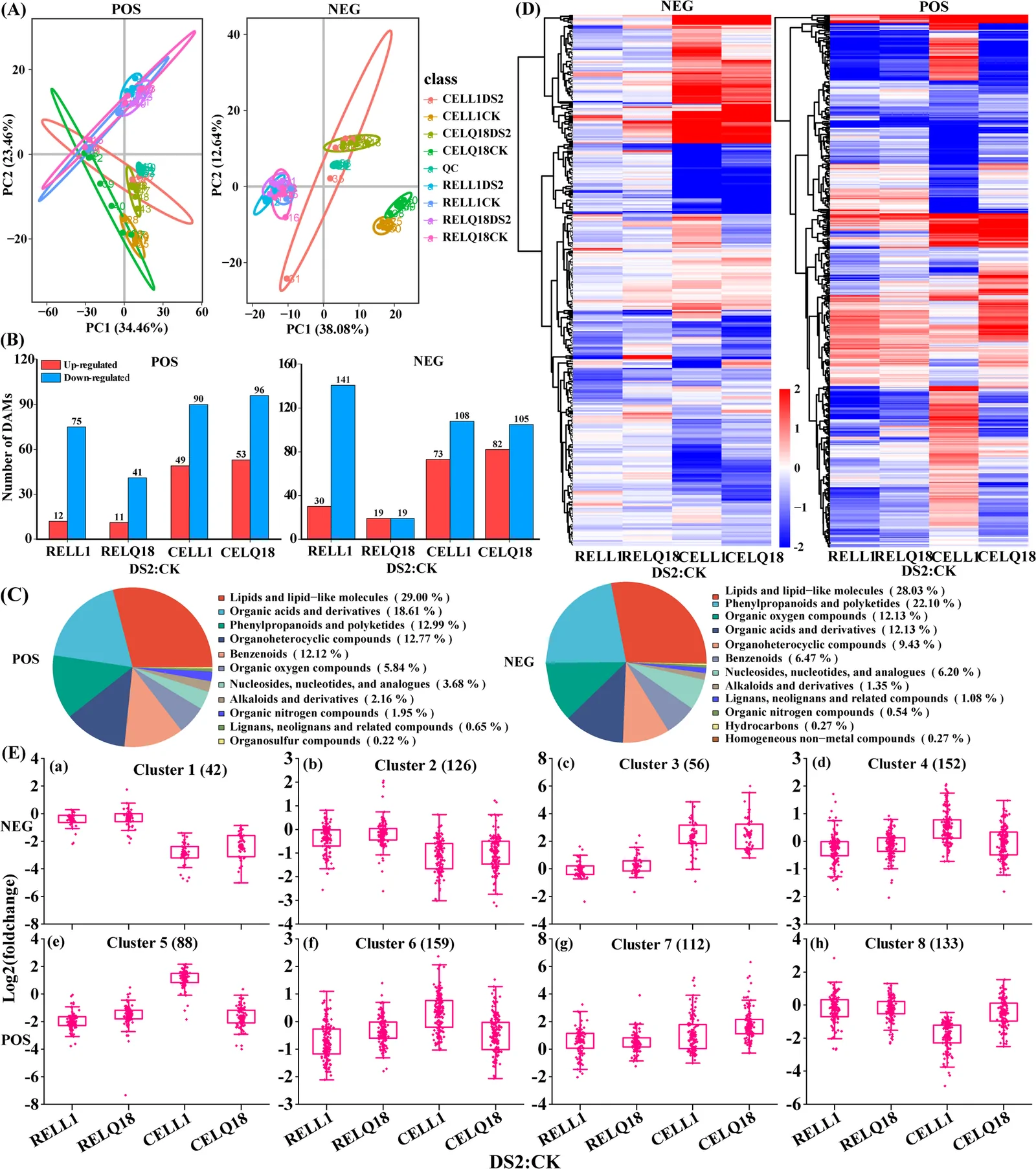
Numbers and categories of differential accumulated metabolites (DAMs) detected in positive ion mode (POS) and negative ion mode (NEG) for quinoa seedlings in DS2 vs. CK at RELL1, RELQ18, CELL1, and CELQ18. **(A)** Principal component analysis of DAMs involved in quinoa seedlings. **(B)** The numbers of the increased and decreased DAMs for quinoa seedlings at different varieties and periods. **(C)** Classification of metabolites and counts in each category. **(D)** *K*-means clustering of DAMs. **(E)** a-h, expression patterns of metabolites specific to different varieties and periods

To comprehensively characterize quinoa seedlings’ metabolic response to drought stress, we conducted untargeted metabolomic analysis (Fig. 3). PCA was then employed to visualize sample distribution patterns in the metabolomic dataset (Fig. 3A). PCA analyses revealed clear separation between developmental stages and between DS2 and CK at cotyledon expansion stage, consistent with the transcriptome results. At radicle emergence, all treatment groups clustered together, indicating limited metabolic response at this stage. Based on quantitative and differential analyses of all assayed metabolites, decreased differential accumulated metabolites (DAMs) were significantly higher than increased DAMs in the pairwise comparison of DS2 vs. CK (Fig. 3B). Metabolomic analysis detected 376 and 492 DAMs in negative and positive ion modes, respectively, which collectively represented major chemical classes such as lipids and lipid-like molecules, phenylpropanoids and polyketides, organic oxygen compounds, organic acids and derivatives, organoheterocyclic compounds, and benzenoids (Fig. 3C). DAMs from both ionization modes were respectively separated into clusters (*k* = 4) using *k*-means analysis in order to further determine the change of DAMs (Fig. 3D). Cluster 3rd in negative and positive modes (56 and 112 DAMs, respectively) was significantly increased at cotyledon expansion and mainly related to carbohydrate (sucrose, maltose, raffinose, stachyose, and trehalose) and phenylpropanoids and polyketides (L-phenylalanine, quercetin, kaempferol, and epigallocatechin) (Fig. 3E-c, E-g). Cluster 1st and clusters 2nd in negative mode was notably decreased at cotyledon expansion and were mainly related to lipids and lipid-like molecules, organic oxygen compounds, organic acids and derivatives, and phenylpropanoids and polyketides (Fig. 3E-a, E-b).

### Integrated transcriptomic and metabolomic analysis under DS2 vs. CK

**Fig. 4:**
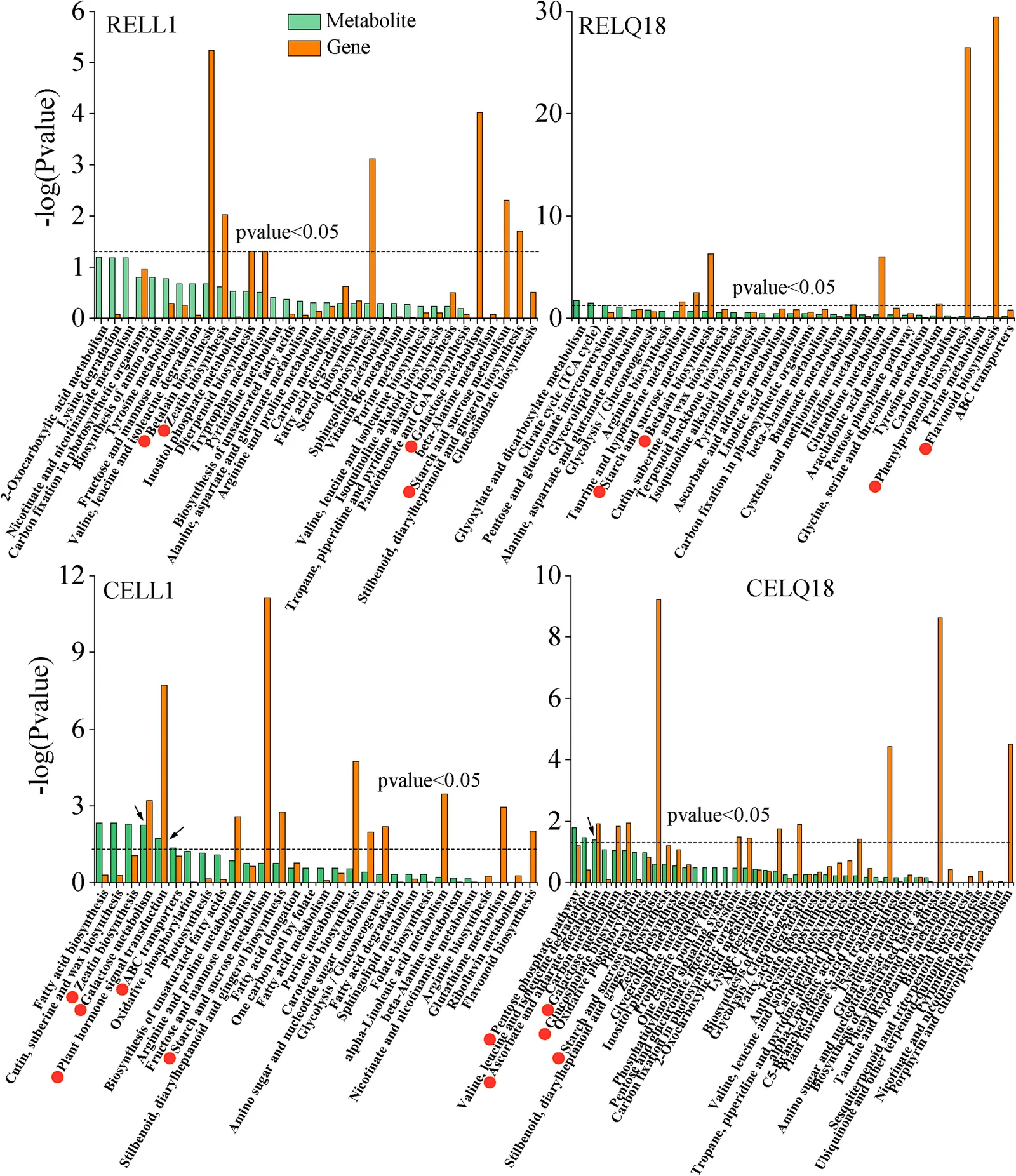
DEGs and DAMs enrichment in KEGG pathways of quinoa seedlings in DS2 vs. CK. Arrows indicate pathways in which both DEGs and DAMs were significantly enriched in the integrated KEGG analysis (*p*value < 0.05). Red dots indicate pathways showing relatively high expression levels of DEGs and DAMs, but without simultaneously reaching statistical significance

To investigate the interplay between DEGs and DAMs in response to drought, we conducted a joint transcriptomic-metabolomic network analysis. A nine-quadrant association analysis was utilized to visualize the correlations between DEGs and DAMs (Supplementary Fig. S3). Only quadrants 3rd and 7th exhibited consistent expression patterns, indicating that the changes in metabolite accumulation might be positively regulated by the genes. In DS2 vs. CK, 285, 78, 322, and 342 metabolites were correlated with 2864, 2444, 8192, and 5641 genes in RELL1, RELQ18, CELL1, and CELQ18, respectively. Integrated KEGG pathway analysis integrating DEGs and DAMs showed that drought-responsive transcripts and metabolites were jointly enriched in multiple metabolic and signaling pathways across the four comparison groups (Fig. 4). Overall, gene-level enrichment signals were generally stronger than metabolite-level enrichment signals. RELQ18 showing particularly strong gene enrichment at the radicle emergence stage, especially in pathways such as phenylpropanoid biosynthesis and flavonoid biosynthesis. Whereas CELL1 and CELQ18 exhibited distinct enrichment patterns at the cotyledon expansion stage. Pathways including starch and sucrose metabolism, galactose metabolism, betalain biosynthesis, and plant hormone signal transduction were among the key enriched pathways identified in the integrated analysis (Fig. 4).

### Carbohydrate metabolism under DS2 vs. CK

**Fig. 5:**
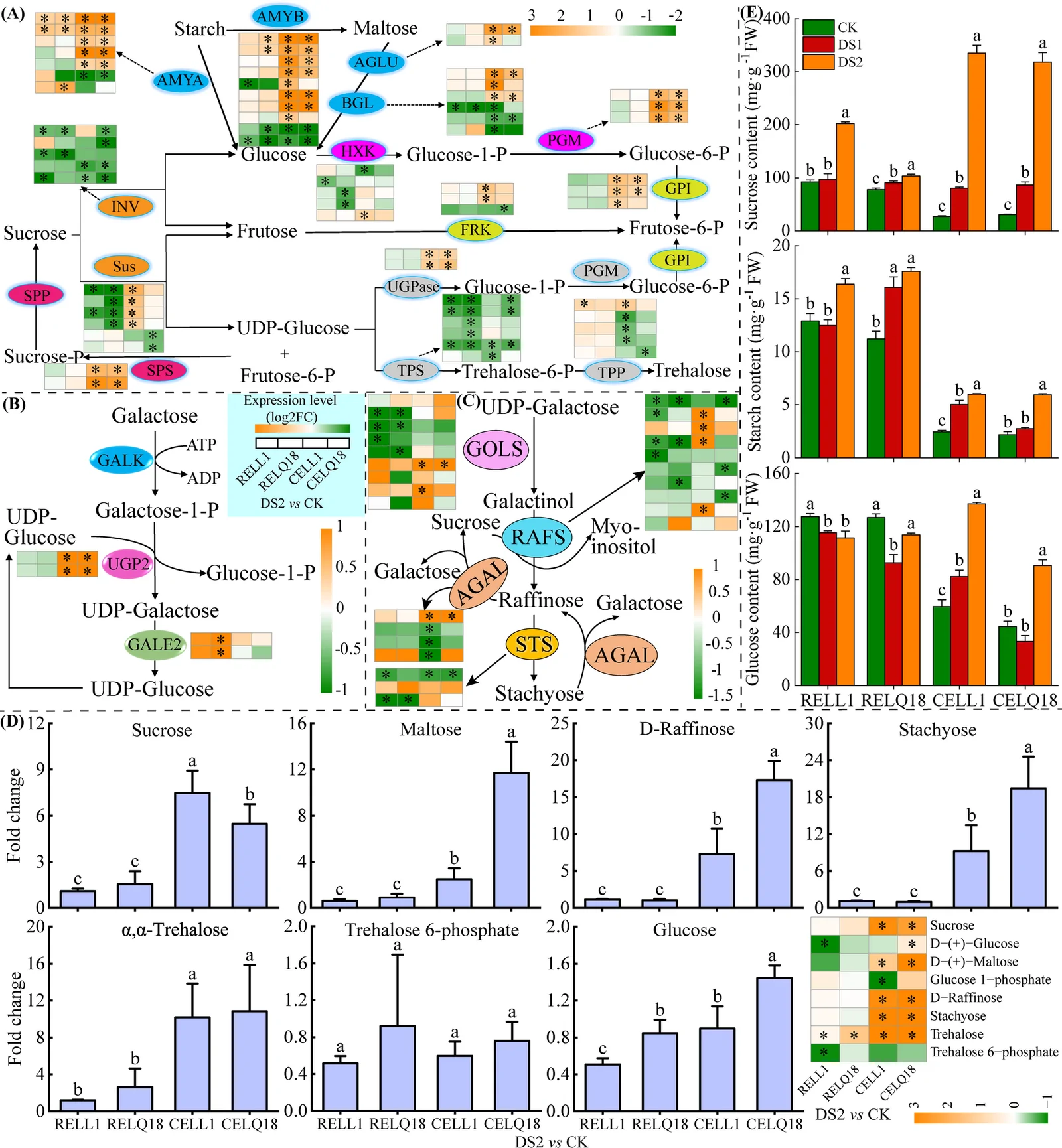
The DEGs and DAMs related to sugar metabolism pathways and the sugar content. **(A)** The DEGs related to the starch and sucrose metabolism. **(B)** DEGs in the metabolism pathway of galactose. **(C)** DEGs in the metabolism pathway of raffinose family oligosaccharides (RFOs). **(D)** The profile of selected sugar metabolites. The data are means of six replicates ± SE. Heatmap shows the relative abundance of selected DAMs. **(E)** Contents of sucrose, glucose, and starch under CK, DS1, and DS2 at RELL1, RELQ18, CELL1, and CELQ18. Different lowercase letters indicate significant differences between drought stress treatments. Bars mean SE (*n* = 3). The asterisk indicates significant changes in DEGs and DAMs. AMYA, alpha-amylase; AMYB, beta-amylase; AGLU, alpha-glucosidase; BGL, beta-glucosidase; Sus, sucrose synthase; INV, invertase; HXK, hexokinase; PGM, phosphoglucomutase; FRK, fructokinase; GPI, glucose-6-phosphate isomerase; SPS, sucrose-phosphate synthase; SPP, sucrose-phosphate phosphatase; TPS, trehalose-phosphate synthase; TPP, trehalose-phosphate phosphatase; UGPase, UTP-glucose-1-phosphate uridylyltransferase; GALK, galactokinase; UGP2, UDP-glucose: galactose-1-phosphate uridylyltransferase; GALE, UDP-glucose 4-epimerase; GOLS, inositol 3-alpha-galactosyltransferase; RAFS, raffinose synthase; AGAL, alpha-galactosidase; STS, stachyose synthetase

Subsequently, we analyzed the DEGs and DAMs involved in starch and sucrose metabolism as well as galactose metabolism in DS2 vs. CK (Fig. 5). Drought stress induced stage-dependent reprogramming of carbohydrate metabolism in quinoa. At the radicle emergence stage, most genes encoding sucrose synthase (*Sus*), invertase (*INV*), and beta-amylase (*AMYB*) were significantly down-regulated, whereas genes encoding alpha-amylase (*AMYA*) were significantly up-regulated under DS2 treatment (Fig. 5A). Genes for beta-glucosidase (*BGL*), hexokinase (*HXK*), and trehalose-phosphate synthase (*TPS*) were also significantly down-regulated under DS2 treatment. Furthermore, the abundance of the DAMs of maltose, glucose, and trehalose 6-phosphate decreased in DS2 vs. CK (Fig. 5D). Then, we measured the content of sucrose, starch, and glucose, and found that glucose content was reduced in the DS2 versus CK comparison (Fig. 5E). Most genes encoding inositol 3-alpha-galactosyltransferase (*GOLS*), raffinose synthase (*RAFS*), and stachyose synthetase (*STS*) were significantly down-regulated (Fig. 5C). However, the abundance of the DAMs of stachyose and raffinose had no significant change (Fig. 5D).

In contrast, at the cotyledon expansion stage, genes associated with starch degradation and sucrose synthesis showed high expression levels and significant up-regulation, whereas those for sucrose degradation and trehalose synthesis were significantly down-regulated (Fig. 5A). Meanwhile, genes encoding phosphoglucomutase (*PGM*), fructokinase (*FRK*), glucose-6-phosphate isomerase (*GPI*), and UTP-glucose-1-phosphate uridylyltransferase (*UGPase*) were significantly up-regulated under the DS2 treatment (Fig. 5A, B). This led to increased sucrose, starch, and glucose content (Fig. 5E). Moreover, most genes for UDP-glucose: galactose-1-phosphate uridylyltransferase (*UGP2*) and *GOLS* were also up-regulated. The abundance of the DAMs of sucrose, maltose, trehalose, stachyose, and raffinose were notably increased under DS2 (Fig. 5D). 

### Hormonal regulation under DS2 vs. CK

**Fig. 6:**
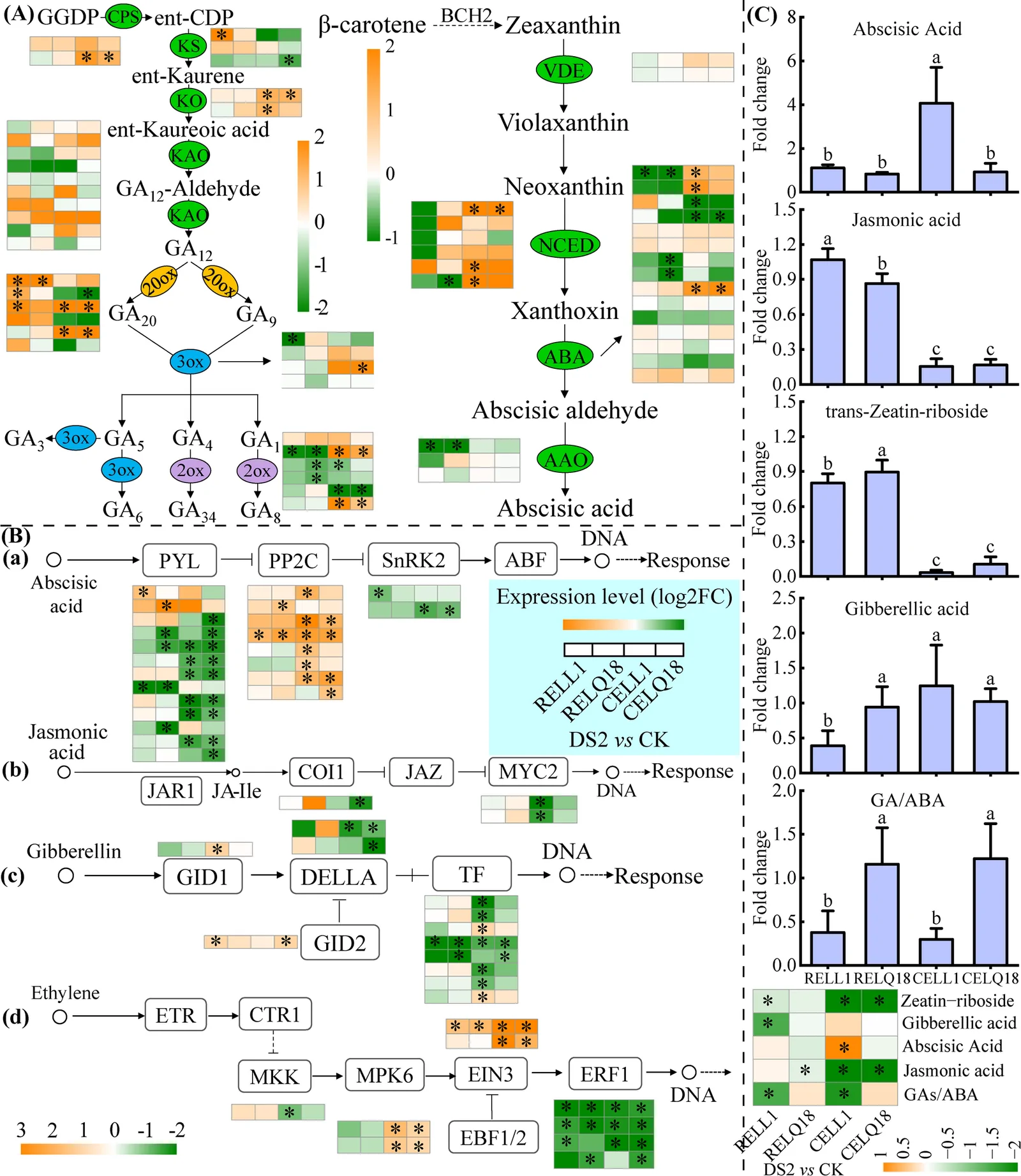
The DEGs and DAMs involved in phytohormones biosynthesis pathway and signal transduction for quinoa seedlings in DS2 vs. CK. **(A)** The DEGs expression level of the genes participated in GAs and ABA biosynthesis pathway. **(B)** The DEGs involved in phytohormones signal transduction. **(C)** The abundance of the DAMs of phytohormones. The data are means of six replicates ± SE. Heatmap shows the relative abundance of selected DAMs. The asterisk indicates significant changes in DEGs and DAMs. CPS, ent-copalyl diphosphate synthase; KS, ent-kaurene synthase; KO, ent-kaurene oxidase; KAO, ent-kaurenoic acid oxidase; 20ox, GA20-oxidase; 3ox, GA3-oxidase; 2ox, GA2-oxidase; BCH2, β-carotene hydroxylase 2; VDE, violaxanthin de-epoxidase; NCED, 9-cis-epoxycarotenoid dioxygenase; ABA, alcohol dehydrogenase; AAO, abscisic-aldehyde oxidase

As central regulators of plant physiology, hormones orchestrate processes ranging from growth to signaling [36], with ABA and GAs being key examples that directly modulate seed germination. The abundance of the DAM of GAs to ABA ratio were notably decreased in LL1 under DS2 at the two stages but slightly increased in LQ18 (Fig. 6C). This suggested that LQ18 is more resistant to drought than LL1. We subsequently analyzed the DEGs involved in ABA metabolism and found that the genes encoding *NCED1* (9-cis-epoxycarotenoid dioxygenase) and *NCED6* were up-regulated, except in LL1 at radicle emergence (Fig. 6A). Concurrently, genes for *CqCYP707A1* and *CqCYP707A2* (cytochrome P450 monooxygenase) were down-regulated (Supplementary Fig. S4). This may reduce ABA catabolism and potentially increase ABA accumulation. Analysis of ABA-related DEGs revealed distinct expression patterns under DS2: most *CqPYL* and *CqSnRK2* genes were markedly down-regulated, whereas *CqPP2C* genes showed up-regulation. (Fig. 6B-a).

Gibberellin biosynthetic gene *CqGA20OX* (gibberellin 20-oxidase) were markedly up-regulated at the two stages. However, genes encoding *CqGA2OX* (gibberellin 2-beta-dioxygenase) and gene for *CqGA3OX* were significantly down-regulated at radicle emergence (Fig. 6A). This led to decreased abundance of the DAMs of GAs, especially for LL1 (Fig. 6C). At cotyledon expansion, gene encoding *CqGA3OX* was significantly up-regulated but genes for *CqGA2OX* exhibiting different results, contributing to increased GAs accumulation (Fig. 6C). In the GA signaling pathway, most associated genes showed down-regulation; in contrast, *GID2* (an F-box protein) was up-regulated (Fig. 6B-c). For ethylene signaling, *CqEIN3* genes were induced, whereas *CqERF1* genes were suppressed (Fig. 6B-d). Most genes related to jasmonic acid (JA) signal transduction were mainly down-regulated under DS2 treatment (Fig. 6B-b). The abundance of the DAMs of JA and zeatin-riboside (ZR) were significantly down-regulated under DS2 treatment (Fig. 6C). 

### Antioxidant system under DS2 vs. CK

**Fig. 7:**
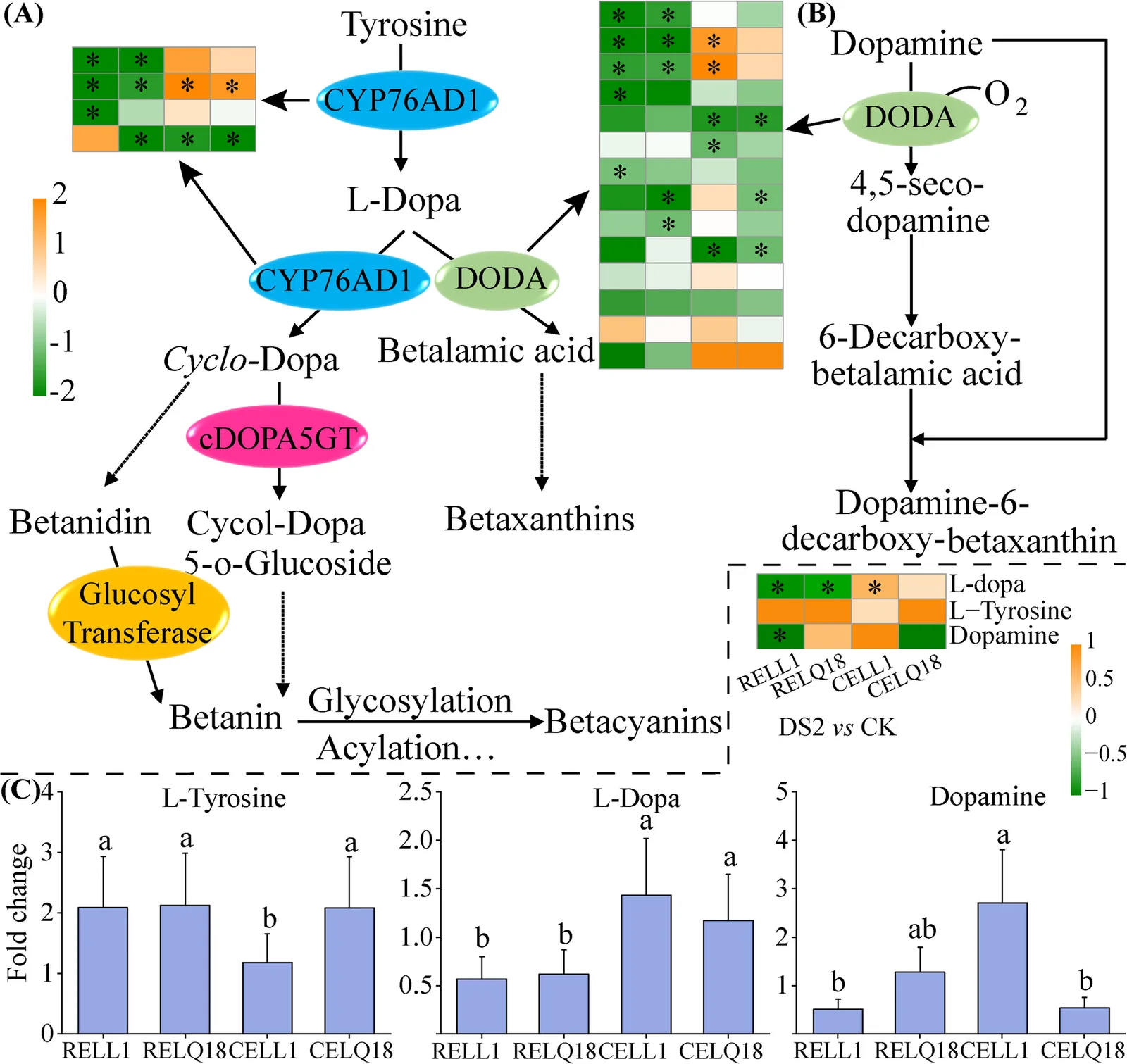
The DEGs and DAMs involved in betalains biosynthesis pathway for quinoa seedlings in DS2 vs. CK. **(A, B)** The DEGs involved in betalains biosynthesis. **(C)** The abundance of the DAMs of levodopa and L-tyrosine. The data are means of six replicates ± SE. Heatmap shows the relative abundance of selected DAMs. The asterisk indicates significant changes in DEGs and DAMs. CYP76AD1, cytochrome P450 enzymes; DODA, 4,5-DOPA-extradiol-dioxygenase; cDOPA5GT, cyclo-DOPA-5-O-glucosyltransferase

The integrated transcriptomic and metabolomic data revealed the central contribution of betalain biosynthesis to drought resistance in quinoa (Fig. 4). In betalain biosynthesis, almost all genes encoding *CYP76AD1* (cytochrome P450 enzymes) and *DODA* (4,5-DOPA-extradiol-dioxygenase) were significantly down-regulated under the DS2 treatment at radicle emergence, but genes encoding *CYP76AD1* were significantly up-regulated at cotyledon expansion (Fig. 7A). The abundance of the DAM of L-Dopa (L-3,4-dihydroxyphenylalanine) was decreased at radicle emergence but increased at cotyledon expansion. In addition, the abundance of the DAM of dopamine was decreased at radicle emergence in LL1 (Fig. 7C).

**Fig. 8:**
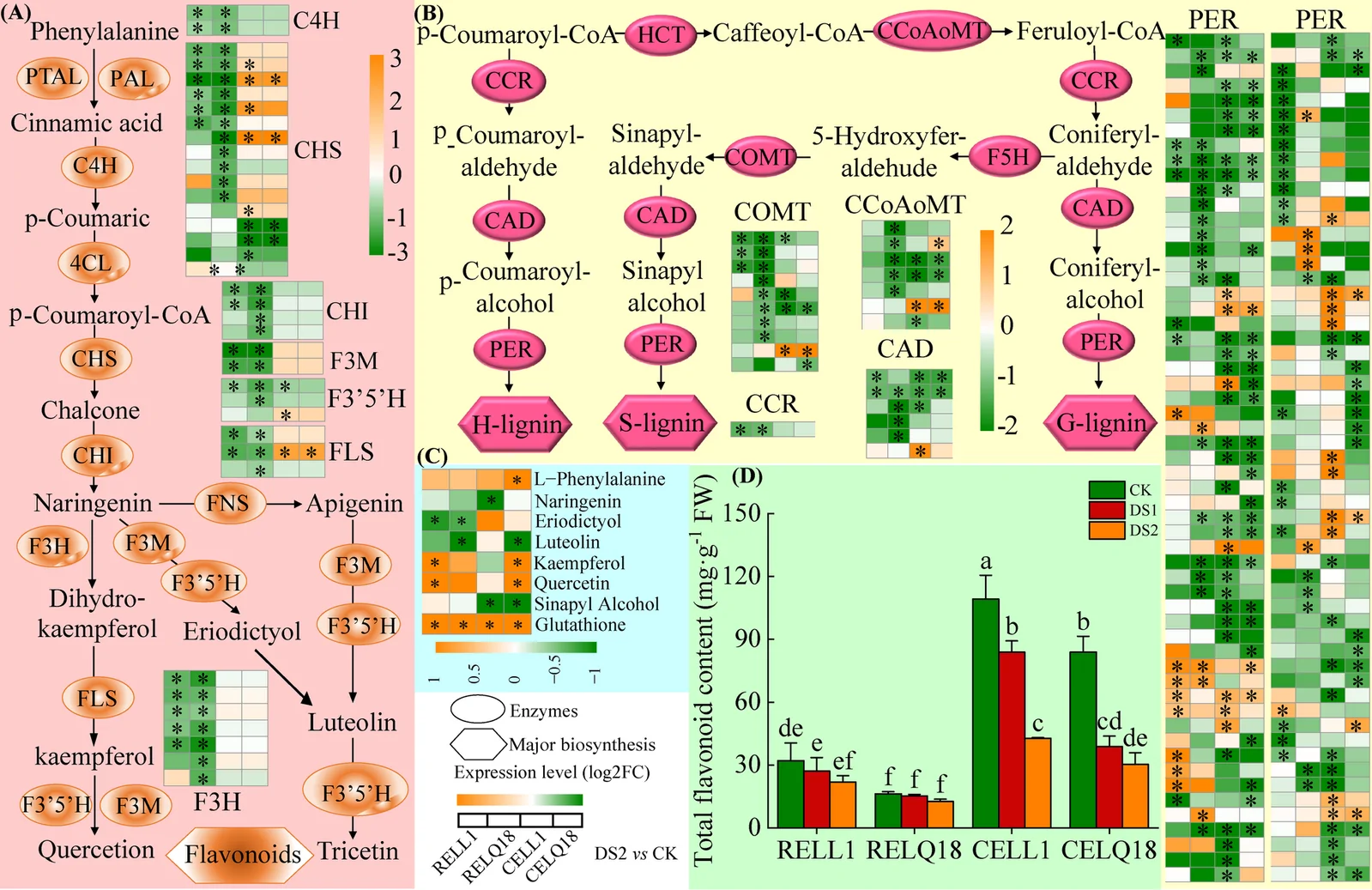
The DEGs and DAMs involved in the biosynthesis of flavonoids and lignin. **(A)** DEGs in the metabolism pathway of flavonoids. **(B)** DEGs in the metabolism pathway of lignin. **(C)** The abundance of the DAMs of secondary metabolites. **(D)** Total flavonoid content. Different lowercase letters indicate significant differences between drought stress treatments. Bars mean SE (*n* = 3). The asterisk indicates significant changes in DEGs and DAMs. PAL, phenylalanine ammonia-lyase; C4H, Cinnamate-4-Hydroxylase; 4CL, 4-Coumarate: Coenzyme A Ligase; CHS, chalcone synthase; CHI, chalcone isomerase; FNS, flavone synthase; F3H, flavanone 3-hydroxylase; F3’5’H, Flavonoid 3’,5’-hydroxylase; F3’H, Flavonoid 3’-hydroxylase; FLS, flavonol synthase; HCT, hydroxycinnamoyl transferase; CCoAOMT, caffeoyl-CoA O-methyltransferase; ‌CCR, cinnamoyl-CoA reductase; COMT, catechol-O-methyltransferase; F5H, Ferulate-5-hydroxylase; CAD, cinnamyl alcohol dehydrogenase; PER, peroxidase

Phenylpropanoid and lignin biosynthesis process were mainly related to cell wall structure, and flavonoid biosynthetic process was associated with Ultraviolet Protector. Under DS2 treatment, most flavonoid biosynthesis genes were significantly down-regulated at radicle emergence. Conversely, homologous genes encoding chalcone synthase (*CHS*) were up-regulated at cotyledon expansion (Fig. 8A), notable as CHS enzyme catalyzes the rate-limiting step in flavonoid biosynthesis. Most of the homologous genes encoding peroxidase (*PER*), cinnamyl alcohol dehydrogenase (*CAD*), catechol-O‐methyltransferase (*COMT*), and caffeoyl-CoA O-methyltransferase (*CCoAoMT*) in P‐lignin, G‐lignin and S‐lignin biosynthesis were also down-regulated in DS2 treatment at both stages (Fig. 8B). Subsequently, flavonoid contents were quantified and shown to be higher at the cotyledon expansion stage compared to radicle emergence. Drought stress did not affect flavonoid content at radicle emergence but significantly reduced it at cotyledon expansion (Fig. 8C).

**Fig. 9:**
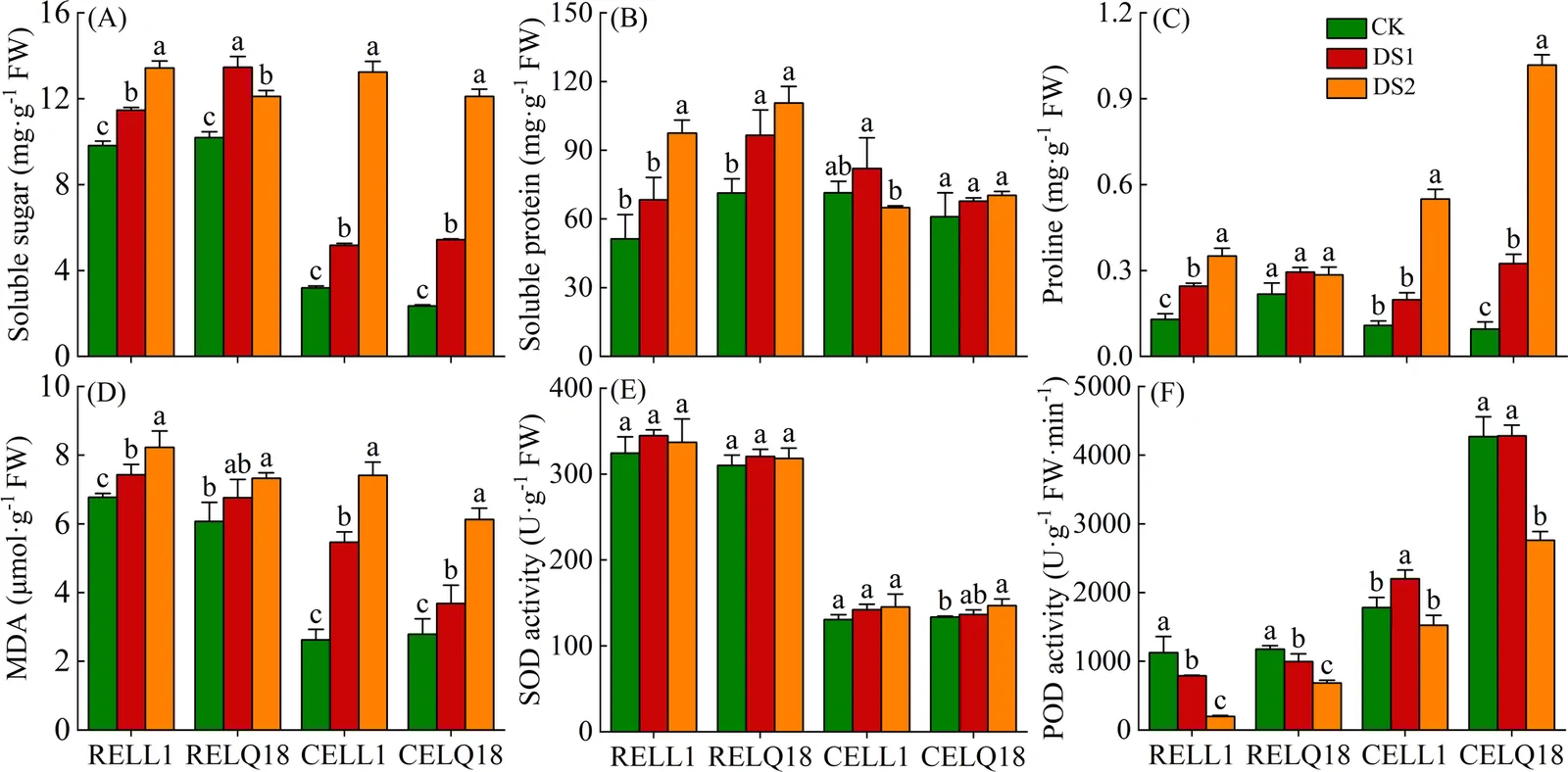
Osmotic adjustment and antioxidant substance contents for quinoa seedlings in the CK (control), DS1 (mild drought stress, 5% PEG-6000) and DS2 (severe drought stress, 20% PEG-6000) treatments at RELL1 (LL1 at radicle emergence), RELQ18 (LQ18 at radicle emergence), CELL1 (LL1 at cotyledon expansion), and CELQ18 (LQ18 at cotyledon expansion). **A** Soluble sugar content. **B** Soluble protein content. **C** Proline content. **D** MDA content. **E** SOD activity. **F** POD activity. *MDA*, malondialdehyde; *SOD*, superoxide dismutase; *POD*, Peroxidase

Moreover, we also examined the content of soluble sugars, antioxidants and osmotic regulators (Fig. 9A-F). The ANOVA indicated that drought stress, genotype, stage, and their interactions all significantly influenced the levels of osmotic adjustment and antioxidant substance (proline exception, Supplementary Table 6). DS1 and DS2 treatments significantly increased the soluble sugars, proline, and malondialdehyde (MDA) levels at both stages and markedly elevated the content of soluble proteins at radicle emergence (Fig. 9A-D). Superoxide dismutase (SOD) activity was not significantly affected by drought stress, but peroxidase (POD) activity decreased under DS2 (Fig. 9E, F). Compared to LL1, LQ18 accumulated more soluble proteins, proline content, and POD activity, and less MDA content under DS1 and DS2 treatments (Fig. 9A-D).

### Verification of RNA sequencing data by qRT-PCR

Eight DEGs from key KEGG-enriched pathways were selected for qRT-PCR analysis to validate the RNA-Seq findings. Selected DEGs exhibited highly consistent expression trends with the sequencing-based FPKM values and further supporting the reliability of the transcriptomic profiles (Supplementary Fig. S5).

## Discussion

### Differential drought tolerance between two quinoa varieties

Both LQ18 and LL1 are drought‑tolerant quinoa varieties, but they exhibited distinct response patterns during seed germination and early seedling establishment under severe drought stress. Under DS2 treatment, LQ18 maintained relatively stable germination indicators, whereas these traits were more markedly reduced in LL1. At the cotyledon expansion stage, severe drought stress inhibited seedling growth in both varieties, but the reduction in radicle length was smaller in LQ18. In addition, LQ18 accumulated higher levels of soluble proteins and proline, retained relatively higher peroxidase (POD) activity, and showed lower malondialdehyde (MDA) content under drought stress, suggesting stronger osmotic adjustment capacity and lower membrane lipid peroxidation. Collectively, these physiological results demonstrate that, LQ18 exhibited a relatively more stable adaptive performance than LL1 under the experimental conditions used in this study.

### Hormonal regulation of germination under severe drought stress

Phytohormones, particularly abscisic acid (ABA) and gibberellins (GAs), play antagonistic roles in regulating seed dormancy and germination across diverse plant species [14, 15]. Under drought stress, ABA rapidly accumulates as a key stress-signaling hormone, inducing seed dormancy and delaying germination to prevent vulnerable seedling emergence under unfavorable conditions. In our study, severe drought stress upregulated the expression of 9-cis-epoxycarotenoid dioxygenase (*NCED*) genes encoding key enzymes in ABA biosynthesis and downregulated cytochrome P450 monooxygenase (*CYP707A*) genes involved in ABA catabolism, consistent with observations in rice and wheat under water deficit conditions [7]. However, the abundance of the DAM of ABA exhibited contrasting patterns between varieties, increasing in LL1 but decreasing in LQ18. This suggests that LQ18 may possess more efficient mechanisms for ABA homeostasis regulation, possibly through enhanced conjugation or compartmentalization, a strategy also observed in drought-tolerant rice cultivars [37]. GAs promote germination by mobilizing stored reserves and enhancing seed coat permeability [14]. Severe drought stress reduced GAs accumulation in both varieties, but the reduction was more pronounced in LL1, leading to a significantly decreased GAs/ABA ratio. This suggesting that LL1 exhibits weak drought resistance than LQ18, and the relative dominance of ABA delays radicle emergence and cotyledon expansion. This phenomenon aligns with the “risk avoidance strategy” of seeds in arid environments, where ABA-mediated prolongation of dormancy prevents germination under subsequent persistent water scarcity.

Beyond ABA and GAs, other hormones such as jasmonic acid (JA) and zeatin riboside (ZR) also play important roles in stress adaptation. JA enhances elongation capacity of radicle tip meristematic cells by activating cell wall-loosening enzymes (e.g., expansins and XTHs), facilitating root system penetration into drought-affected soil for water acquisition, while cytokinins regulate cell division and meristem activity [38]. The lower JA and ZR accumulation levels under severe drought stress, particularly in LL1, likely contributed to the impaired radicle elongation and overall growth suppression.

### Stage-specific reprogramming of carbohydrate metabolism

Carbohydrate metabolism undergoes dynamic changes during seed germination and early seedling growth, providing both energy and carbon skeletons for developing tissues [7, 8]. Our integrated transcriptomic and metabolomic analyses revealed stage-specific reprogramming of starch, sucrose, and raffinose family oligosaccharide metabolism in response to severe drought stress. At the radicle emergence stage, severe drought stress suppressed the expression of genes encoding sucrose synthase, invertase, and beta-amylase, while upregulating alpha-amylase. This expression pattern resulted in reduced levels of glucose and maltose, despite increased starch content. The decrease in glucose affects the provision of energy (ATP) through glycolysis (EMP) and the tricarboxylic acid cycle (TCA), prolonging radicle emergence. Similar glucose limitation under drought stress has been observed in Arabidopsis seeds, where low glucose levels suppress ABA inhibition specifically at the radicle emergence stage. Whereas ABA inhibition at seedling growth is suppressed by sugar allocation and the modulation of sugar metabolism [39, 40]. This may explain the non-significant change in ABA abundance at radicle emergence under drought stress. Additionally, as a central regulator of cell growth control, TOR (target of rapamycin) kinase activation promotes ribosome biosynthesis and cell cycle proteins expression, accelerating cell division [41, 42]. Low glucose level significantly inhibited *CqTOR* kinase gene expression (Supplementary Fig S6), delayed both cell division and radicle elongation, reduced germination indicators, especially in LL1.

In contrast, at the cotyledon expansion stage, severe drought stress induced the expression of genes involved in starch degradation and sucrose synthesis, leading to increased glucose, sucrose, and starch content. This shift likely reflects a transition from heterotrophic to autotrophic metabolism, where the expanding cotyledons begin to photosynthesize while still relying on stored reserves. The increased glucose was primarily utilized for synthesizing osmo-protectants (e.g., proline and glycine betaine), sustaining basic respiratory metabolism (ATP supply), and activating stress-responsive genes like late embryogenesis abundant proteins (*LEA*) and heat shock proteins (*HSP*) [43–45].

Raffinose family oligosaccharides (RFOs), primarily including raffinose and stachyose, function as osmo-protectants and membrane stabilizers under abiotic stress [46]. In our study, severe drought stress increased the abundance of raffinose and stachyose at the cotyledon expansion stage, coinciding with the upregulation of genes encoding galactinol synthase, raffinose synthase, and stachyose synthase. This accumulation pattern is consistent with findings in chickpea and cyclocarya paliurus, where raffinose family oligosaccharides accumulate under drought stress to protect cellular membranes and scavenge reactive oxygen species [12, 13].

### Antioxidant and osmotic adjustment mechanisms

Reactive oxygen species (ROS) overaccumulation is a primary consequence of drought stress, causing oxidative damage to cellular components [18, 47]. To counteract this, plants employ both enzymatic and non-enzymatic antioxidant systems to mitigate oxidative stress [18, 19, 22]. In our study, superoxide dismutase (SOD) activity was not significantly affected by drought stress, while peroxidase (POD) activity significantly decreased, indicating that enzymatic antioxidant capacity may be insufficient under extreme water deficit. Soluble sugars, proteins, and proline are crucial osmotic adjustment substances and nutrients. Under drought stress, plants elevate osmotic adjustment levels to maintain cellular turgor pressure, increase water retention and uptake, and mitigate stress damage [48, 49]. LQ18 accumulated more soluble proteins and proline, less MDA content, and higher POD activity, suggesting more effective osmotic adjustment and oxidative stress mitigation.

Non-enzymatic antioxidants also contributed to drought tolerance. Glutathione, a major cellular antioxidant, accumulated under severe drought, particularly in LQ18. This observation aligns with studies in barley and Arabidopsis, where glutathione plays a critical role in abscisic acid signaling and oxidative stress defense [19, 20]. Betalains, which are unique to the Caryophyllales order including quinoa, also showed stage-specific accumulation patterns, with reduced levodopa (L-Dopa) levels at radicle emergence but increased levels at cotyledon expansion. The antioxidant properties of betalains have been well documented, and their accumulation under stress may contribute to photoprotection and reactive oxygen species scavenging [21, 50].

Interestingly, while flavonoid biosynthesis is often upregulated under abiotic stress in many plant species [25], our study showed downregulation of flavonoid-related genes and reduced total flavonoid content under severe drought. This contrasts with findings in tomato and foxtail millet, where drought stress typically induces flavonoid accumulation [51, 52]. The divergent response may reflect the severe level of stress imposed, or may indicate that quinoa prioritizes other protective mechanisms such as raffinose family oligosaccharides and betalain accumulation under extreme water deficit.

## Conclusion

Through a comprehensive phenotypic, physiological, transcriptomic, and metabolomic analysis of drought stress, we have revealed response mechanisms to drought stress. Our results indicated that severe drought stress notably impaired germination and seedling establishment, especially for LL1. Multi-omics integration revealed that drought tolerance involves dynamic, stage-specific metabolic reprogramming. The suppression of starch and sucrose and galactose metabolism pathways at radicle emergence, contrasted by their induction at cotyledon expansion, modulated sugar availability and influenced GAs/ABA homeostasis. Furthermore, enhanced tolerance in LQ18 under severe drought stress was strongly associated with the effective upregulation of antioxidant systems and significant accumulation of key osmo-protectants and protective metabolites. These findings advance the mechanistic understanding of drought tolerance in early quinoa development and serve as a valuable resource for enhancing crop resilience in water-limited settings.

## Supplementary Information

Supplementary Material 1.

Supplementary Material 2.

Supplementary Material 3.

## Data Availability

The data supporting the differentially expressed genes (DEGs) and differentially abundant metabolites (DAMs) between the DS2 and CK are available in Supplementary Tables S1–S17. The RNA-seq metadata are available in the NCBI repository under the accession number PRJNA1443003.
