# Supplementary material for: Drought disrupts quinoa seed germination and seedling establishment via sugar metabolism and hormonal perturbations
Source: BMC Plant Biol. 2026 Jun 30;26:1604. doi: 10.1186/s12870-026-09410-z (PMC13584531; doi:10.1186/s12870-026-09410-z)
Supplement: Supplementary file 1 [file 12870_2026_9410_MOESM1_ESM.docx]

**Table 1.** Effect of water stress on germination rate of quinoa seeds LL1.

| Treatment | Germination number | | | | | Germination rate (%) |
| --- | --- | --- | --- | --- | --- | --- |
|  | 12 h | 24 h | 36 h | 48 h | 72 h |  |
| **Control** | **56.3 a** | **88.7 a** | **92.7 a** | **95.3 ab** | **95.3 ab** | **95.3 ab** |
| 2.25% | 52.0 a | 86.3 a | 92.0 ab | 96.0 a | 96.0 ab | 96.0 ab |
| **5%** | **41.3 b** | **80.7 ab** | **91.7 ab** | **94.3 ab** | **97.0 a** | **97.0 a** |
| 10% | 21.7 c | 75.7 b | 87.3 ab | 90.0 bc | 92.7 b | 92.7 b |
| 15% | 8.0 d | 75.0 b | 84.7 b | 88.0 c | 92.3 b | 92.3 b |
| **20%** | **1.0 e** | **24.3 c** | **40.0 c** | **50.7 d** | **80.3 c** | **80.3 c** |

**Table 2.** RNA integrity (RIN) values of all RNA-seq samples used for transcriptome sequencing

| No. | Sample name | Stage | Genotype | Treatment | Replicate | RIN |
| --- | --- | --- | --- | --- | --- | --- |
| 1 | RELL1 CK-1 | Radicle emergence stage | LL1 | CK | 1 | 9.8 |
| 2 | RELL1 CK-2 | Radicle emergence stage | LL1 | CK | 2 | 9.7 |
| 3 | RELL1 CK-3 | Radicle emergence stage | LL1 | CK | 3 | 9.8 |
| 4 | RELL1 DS2-1 | Radicle emergence stage | LL1 | DS2 | 1 | 9.5 |
| 5 | RELL1 DS2-2 | Radicle emergence stage | LL1 | DS2 | 2 | 9.8 |
| 6 | RELL1 DS2-3 | Radicle emergence stage | LL1 | DS2 | 3 | 8.1 |
| 7 | RELQ18 CK-1 | Radicle emergence stage | LQ18 | CK | 1 | 9.3 |
| 8 | RELQ18 CK-2 | Radicle emergence stage | LQ18 | CK | 2 | 9.8 |
| 9 | RELQ18 CK-3 | Radicle emergence stage | LQ18 | CK | 3 | 9.8 |
| 10 | RELQ18 DS2-1 | Radicle emergence stage | LQ18 | DS2 | 1 | 9.6 |
| 11 | RELQ18 DS2-2 | Radicle emergence stage | LQ18 | DS2 | 2 | 9.0 |
| 12 | RELQ18 DS2-3 | Radicle emergence stage | LQ18 | DS2 | 3 | 9.8 |
| 13 | CELL1 CK-1 | Cotyledon expansion stage | LL1 | CK | 1 | 8.7 |
| 14 | CELL1 CK-2 | Cotyledon expansion stage | LL1 | CK | 2 | 9.0 |
| 15 | CELL1 CK-3 | Cotyledon expansion stage | LL1 | CK | 3 | 8.8 |
| 16 | CELL1 DS2-1 | Cotyledon expansion stage | LL1 | DS2 | 1 | 9.1 |
| 17 | CELL1 DS2-2 | Cotyledon expansion stage | LL1 | DS2 | 2 | 8.7 |
| 18 | CELL1 DS2-3 | Cotyledon expansion stage | LL1 | DS2 | 3 | 9.1 |
| 19 | CELQ18 CK-1 | Cotyledon expansion stage | LQ18 | CK | 1 | 8.6 |
| 20 | CELQ18 CK-2 | Cotyledon expansion stage | LQ18 | CK | 2 | 9.1 |
| 21 | CELQ18 CK-3 | Cotyledon expansion stage | LQ18 | CK | 3 | 9.0 |
| 22 | CELQ18 DS2-1 | Cotyledon expansion stage | LQ18 | DS2 | 1 | 9.2 |
| 23 | CELQ18 DS2-2 | Cotyledon expansion stage | LQ18 | DS2 | 2 | 9.3 |
| 24 | CELQ18 DS2-3 | Cotyledon expansion stage | LQ18 | DS2 | 3 | 9.1 |

**Table 3.** The primer designed for RT-qPCR.

| Gene | Forward primer | Reverse primer |
| --- | --- | --- |
| *AMYA* | CAACCAAGACGGACCGAGAA | GCAGCAGCTTGAAGAACACC |
| *AMYB* | TGTCACTATCCCCCTCCCTG | CATCTGGATAGGTGTCCGGC |
| *GOLS* | AGAGACTCCTCCCACTTGCT | CAGTCGCGCAATAGTGCATC |
| *GA2OX* | CAAGACAGCAATCGCGGAAG | GTGTAGGAGAACCCCAACGG |
| *NCED* | TGATGGTATGATCCACGCCG | ATGGGTCTACCCAAAGCACG |
| *CYP76AD1* | CGACGTCGATTGGCGTCTAA | ATCATCGGTCGTCTTCGTCG |
| *DODA* | GTTCCGGAAGTGCAACACAC | AGTGAGAGCAGTGTCAAGCC |
| *CHS* | CCACTGAGACCCACTTGGAC | CTTCTCGCAAATGCCCATCG |
| *Actin-1* | TTCCCAAGTATTGTTGGTCGTC | CCAGATCTTCTCCATGTCATCC |

Note: Amplification efficiency values were not available because Bio-Rad CFX Maestro software (version 2.2) did not output efficiency values under the routine analysis settings used in this study.

**Table 4.** Sources of variability for quinoa germination and seedlings growth.

| Variable | Factor | *F, P*-value |
| --- | --- | --- |
| Germination rate | (WS) Water stress | 60.0*** |
|  | (G) Genotype | 60.6*** |
|  | WS*G | 23.0*** |
| Germinative force | (WS) Water stress | 7.9* |
|  | (G) Genotype | 109.0*** |
|  | WS*G | 128.6*** |
| Germination index | (WS) Water stress | 38.4*** |
|  | (G) Genotype | 154.1*** |
|  | WS*G | 143.4*** |
| Overall length | (WS) Water stress | 78.3*** |
|  | (G) Genotype | 84.0*** |
|  | WS*G | 10.2** |
| Seedlings height | (WS) Water stress | 37.9*** |
|  | (G) Genotype | 145.7*** |
|  | WS*G | 2.2ns |
| Hypocotyl length | (WS) Water stress | 111.2*** |
|  | (G) Genotype | 120.9*** |
|  | WS*G | 11.5** |
| Radicle length | (WS) Water stress | 44.2*** |
|  | (G) Genotype | 21.0*** |
|  | WS*G | 10.4** |
| Fresh weight | (WS) Water stress | 28.8*** |
|  | (G) Genotype | 41.8*** |
|  | WS*G | 1.4ns |
| Dry weight | (WS) Water stress | 9.7** |
|  | (G) Genotype | 5.9* |
|  | WS*G | 0.4ns |
| Water content | (WS) Water stress | 1.9ns |
|  | (G) Genotype | 17.5*** |
|  | WS*G | 0.1ns |

**Table 5.** Sources of variability for quinoa sugar, osmotic adjustment, and antioxidant substance contents.

| Variable | Factor | *F, P*-value | Variable | Factor | *F, P*-value |
| --- | --- | --- | --- | --- | --- |
| Sucrose | (WS) Water stress | 21.4*** | Proline | (WS) Water stress | 1.3ns |
|  | (G) Genotype | 603.9*** |  | (G) Genotype | 6.5** |
|  | (S) Stage | 60.7*** |  | (S) Stage | 1.8ns |
|  | WS*G | 15.8*** |  | WS*G | 0.3ns |
|  | WS*S | 16.5*** |  | WS*S | 0.8ns |
|  | G*S | 233.7*** |  | G*S | 3.2ns |
|  | W*G*S | 5.8** |  | W*G*S | 1.0ns |
| Starch | (WS) Water stress | 0.1ns | MDA | (WS) Water stress | 43.2*** |
|  | (G) Genotype | 73.0*** |  | (G) Genotype | 142.4*** |
|  | (S) Stage | 1274.9*** |  | (S) Stage | 338.7*** |
|  | WS*G | 3.4* |  | WS*G | 5.2* |
|  | WS*S | 10.7** |  | WS*S | 0.6ns |
|  | G*S | 1.6ns |  | G*S | 35.7*** |
|  | W*G*S | 13.4*** |  | W*G*S | 4.8* |
| Glucose | (WS) Water stress | 91.2*** | SOD | (WS) Water stress | 5.4* |
|  | (G) Genotype | 70.1*** |  | (G) Genotype | 3.5* |
|  | (S) Stage | 301.5*** |  | (S) Stage | 1982.5*** |
|  | WS*G | 12.2*** |  | WS*G | 0.4ns |
|  | WS*S | 42.0*** |  | WS*S | 5.0* |
|  | G*S | 93.1*** |  | G*S | 0.7ns |
|  | W*G*S | 4.8* |  | W*G*S | 0.0ns |
| Soluble sugar | (WS) Water stress | 1.4ns | POD | (WS) Water stress | 91.1*** |
|  | (G) Genotype | 1433.7*** |  | (G) Genotype | 21.0*** |
|  | (S) Stage | 2691.1*** |  | (S) Stage | 298.8*** |
|  | WS*G | 123.1*** |  | WS*G | 1.1ns |
|  | WS*S | 24.5*** |  | WS*S | 54.3*** |
|  | G*S | 675.2*** |  | G*S | 2.9ns |
|  | W*G*S | 44.0*** |  | W*G*S | 4.7* |
| Soluble protein | (WS) Water stress | 6.9* | Flavonoid | (WS) Water stress | 108.7*** |
|  | (G) Genotype | 15.3*** |  | (G) Genotype | 102.5*** |
|  | (S) Stage | 23.8*** |  | (S) Stage | 528.2*** |
|  | WS*G | 4.5* |  | WS*G | 7.1** |
|  | WS*S | 25.5*** |  | WS*S | 16.0*** |
|  | G*S | 19.4*** |  | G*S | 65.2*** |
|  | W*G*S | 9.4*** |  | W*G*S | 5.7** |
