# Supplementary material for: Drought disrupts quinoa seed germination and seedling establishment via sugar metabolism and hormonal perturbations
Source: BMC Plant Biol. 2026 Jun 30;26:1604. doi: 10.1186/s12870-026-09410-z (PMC13584531; doi:10.1186/s12870-026-09410-z)
Supplement: Supplementary file 2 [file 12870_2026_9410_MOESM3_ESM.docx]

**Figure S1.** The Pearson correlations between samples and PCA analysis. RELL1, LL1 at radical emergence; RELQ18, LQ18 at radical emergence; CELL1, LL1 at cotyledon expansion; CELQ18, LQ18 at cotyledon expansion.

**Figure S2.** Gene Ontology (GO) and Kyoto Encyclopedia of Genes and Genomes (KEGG) enrichment analysis of the overlapped differentially expressed genes (DEGs). Red-highlighted pathway names indicate the key significantly enriched pathways emphasized in the main text and discussion.

**Figure S3.** Nine-quadrant map of genes and metabolites at RELL1, RELQ18, CELL1, and CELQ18.

**Figure S4.** DEGs for *CqCYP707A* (cytochrome P450 monooxygenase) under the DS2 and CK treatments at RELL1, RELQ18, CELL1, and CELQ18.

**Figure S5.** RT-qPCR validation of transcriptome data for eight selected genes. Bars represent relative expression levels determined by RT-qPCR (shown as mean ± SE, n = 3), and lines represent the corresponding RNA-seq expression trends. Relative RT-qPCR expression levels were calculated using the 2^−ΔΔCt^ method.

**Figure S6.** DEGs for TOR (target of rapamycin) under the DS2 and CK treatments at RELL1, RELQ18, CELL1, and CELQ18.
